# Impact of Transition Metal Layer Vacancy on the Structure and Performance of P2 Type Layered Sodium Cathode Material

**DOI:** 10.1007/s40820-024-01439-9

**Published:** 2024-07-08

**Authors:** Orynbay Zhanadilov, Sourav Baiju, Natalia Voronina, Jun Ho Yu, A-Yeon Kim, Hun-Gi Jung, Kyuwook Ihm, Olivier Guillon, Payam Kaghazchi, Seung-Taek Myung

**Affiliations:** 1 Department of Nanotechnology and Advanced Materials Engineering and Sejong Battery Institute, Hybrid Materials Research Center, Sejong University, 98 Gunja-Dong, Gwangjin-Gu, Seoul, 05006 South Korea; 2 Institute of Energy and Climate Research-Materials Synthesis and Processing (IEK-1), Forschungszentrum Jülich GmbH, 52425 Jülich, Germany; 3 Center for Energy Storage Research, Korea Institute of Science and Technology, Seoul, 02792 South Korea; 4 KIST-SKKU Carbon-Neutral Research Center, Sungkyunkwan University, Suwon, 16419 South Korea; 5 Department of Energy Science, Sungkyunkwan University, Suwon, 16419 South Korea; 6 Pohang Accelerator Laboratory, 80 Jigokro-127-Beongil, Nam-Gu, Pohang, Gyeongbuk 37673 South Korea; 7 MESA+ Institute for Nanotechnology, University of Twente, 7500 AE Enschede, The Netherlands

**Keywords:** Layered oxide, Oxygen evolution, Sodium battery, Vacancy, Cathode

## Abstract

This study explores the impact of introducing vacancy in the transition metal layer of rationally designed Na_0.6_[Ni_0.3_Ru_0.3_Mn_0.4_]O_2_ (NRM) cathode material. The incorporation of Ru, Ni, and vacancy enhances the structural stability during extensive cycling, increases the operation voltage, and induces a capacity increase while also activating oxygen redox, respectively, in Na_0.7_[Ni_0.2_V_Ni0.1_Ru_0.3_Mn_0.4_]O_2_ (V-NRM) compound. Various analytical techniques including transmission electron microscopy, X-ray absorption near edge spectroscopy, *operando* X-ray diffraction, and *operando* differential electrochemical mass spectrometry are employed to assess changes in the average oxidation states and structural distortions. The results demonstrate that V-NRM exhibits higher capacity than NRM and maintains a moderate capacity retention of 81% after 100 cycles. Furthermore, the formation of additional lone-pair electrons in the O 2*p* orbital enables V-NRM to utilize more capacity from the oxygen redox validated by density functional calculation, leading to a widened dominance of the OP4 phase without releasing O_2_ gas. These findings offer valuable insights for the design of advanced high-capacity cathode materials with improved performance and sustainability in sodium-ion batteries.

**Supplementary Information:**

The online version contains supplementary material available at 10.1007/s40820-024-01439-9.

## Highlights

- Vacancy in the transition metal layer of sodium cathode material induces the formation lone-pair electrons in the O 2*p* orbital.
- Material delivers more capacity from the oxygen redox validated by density functional calculation.
- Widened dominance of the OP4 phase without releasing O_2_ gas.

**Supplementary Information:**

The online version contains supplementary material available at 10.1007/s40820-024-01439-9.

## Graphical abstract

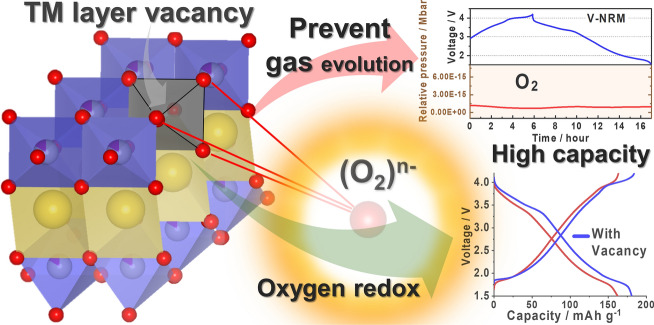

## Introduction

Increasing environmental concerns and global warming have led to an urgent need to reduce the use of fossil fuels. Renewable energy supported by sustainable energy storage is considered the most plausible alternative to conventional power generation, thus highlighting the importance of energy storage systems (ESSs). Lithium-ion batteries (LIBs) have been adopted as one of the most appropriate ESSs to ensure high energy density and long-term cycling. Therefore, lithium resources are critical to provide LIB-assisted power sources from portable to stationary applications. The uneven distribution and soaring price of lithium have led to the search for an alternative power system in terms of economic factors. Sodium is one of the most abundant elements on the Earth’s crust, such that the use of sodium ions has a great advantage in lowering the cost of ESSs [1–3]. However, the corresponding energy density of sodium-ion batteries (SIBs) is lower than that of LIBs due to the low potential of sodium versus the standard hydrogen electrode (SHE), namely, Na^+^/Na: − 2.71 V and Li^+^/Li: − 3.04 V [2, 4]. Therefore, cathode materials of SIBs need to have high capacity to compensate for the low energy density, which can be compatible with the energy density of LIBs.

Among existing cathode materials, layered sodium transition metal oxides Na_x_TMO_2_ (0 < x ≤ 1) with the configuration Na–O–A (A: Li [5, 6], Mg [7–9], Zn [10–12], and vacancy [13–16]) are of interest because of their peculiar behavior to show reversible oxygen redox in addition to the redox of transition metals in Na cells; thus, additional capacity is delivered by the oxygen redox [17–31]. It is known that the A elements, particularly Li, undergo migration toward Na layers on desodiation (charge), which spontaneously produces unpaired electrons in the O 2*p* orbital to trigger the oxidation of O^2−^ toward dimerized (O_2_)^n−^ (*n* < 2) in the oxide lattice, and vice versa on sodiation (discharge). Another condition to facilitate oxygen redox is that the lattice oxygen can be evolved from the structure at a highly desodiated state, which leaves electron holes that simultaneously induce the extraction of Na^+^ ions for charge compensation on desodiation [32]. The corresponding structure is reorganized on the subsequent sodiation, for which the main redox reaction progresses via the reduction of transition metal (TM) components. It is of interest to observe the effect of vacancies in TM layers on the oxygen redox [33], which accelerate the oxygen redox reaction by forming the Na–O–V (V: vacancy) configuration that spontaneous forms unpaired electrons in the oxygen orbital [34, 35]. The oxidation of oxygen derived from the vacancy is typically initiated at ~ 4 V with an additional voltage plateau, although the contribution is minor compared with the main reaction triggered by the migration of A from the TM to Na layers in the Na–O–A configuration. Density functional theory (DFT) calculation by Yang et al. [34] demonstrated that the oxygen close to the vacancies (Na–O–V) interacts with more charge than the oxygen coordinated with A in Na–O–A on charge; hence, more charge compensation with the vacancy is responsible for assisting more capacity. These behaviors are demonstrated in P2-Na_0.63_[Mg_0.143_Mn_0.820_V_Mg0.036_]O_2_ [34] and Na_2/3_[Mg_1/9_Mn_7/9_V_Mg1/9_]O_2_ [35].

The electrochemical activity is dependent on the redox potential of Mn^4+^/Mn^3+^ and O^2−^/(O_2_)^n−^ (*n* < 2) redox pairs for the above cathode materials with vacancies, such that the material experiences a large hysteresis on operation voltage between charge and discharge with slow kinetics and low operation voltage. In terms of charge compensation, the Mn and O redox pairs are sufficient to have high capacity; however, elaboration is required to raise the operation voltage. We confirmed that utilization of the Ni^4+^/Ni^2+^ redox pair greatly raises the operation voltage of the oxygen redox above 3.6 V on discharge for P2-Na_2/3_[Zn_0.15_Ni_0.15_Mn_0.7_]O_2_ in addition to the improved electric conductivity with the help of the Ni component [18]. In addition, Mn is the essential element facilitating the oxygen reaction in the structural framework, although the corresponding redox potential of Mn^4+^/Mn^3+^ is empirically observed between 1.5 and 2.5 V in SIBs. Deep discharge induces the formation of Mn^3+^, which undergoes Jahn–Teller distortion such that the *z*-axis of the Mn^3+^O_6_ octahedron is undesirably elongated. This phenomenon is also unfavorable for long-term capacity retention because the repetitive change in the *z*-axis reduces the structural integrity during extensive cycling. This effect thus necessitates partial replacement of Mn with other TM elements that possess sufficient bonding strength with oxygen, which mitigates the abnormal Mn–O bond length along the *z*-axis. Therefore, it is favored to adopt a TM element with equivalent oxidation state (4 +) to satisfy the charge neutrality in the TM layers. Among the possible candidates showing redox reaction (e.g., Ti, V, Ru), Ru is of interest in terms of redox potential and bond strength with covalency.

Based on the above hypothesis, we rationally designed Na_0.6_[Ni_0.3_Ru_0.3_Mn_0.4_]O_2_ (NRM) and vacancy introduced Na_0.7_[Ni_0.2_V_Ni0.1_Ru_0.3_Mn_0.4_]O_2_ (V-NRM) compounds, for which the average oxidation state of Mn was controlled to be 4 + . The introduction of vacancies in the TM layer increased the sodium content from 0.6 to 0.7 mol and raised the average oxidation state of Ni higher than ~ 2 + . Correspondingly, a slight improvement in the capacity including rate performance was observed for V-NRM (~ 184 mAh g^−1^ from ~ 163 mAh g^−1^ for NRM). An interesting feature is the emergence of an additional short voltage plateau at ~ 3.9 V on charge and at ~ 3.8 V on discharge for V-NRM. Both compounds underwent a simple phase transition from P2 to OP4 during de/sodiation, as confirmed by *operando* X-ray diffraction (*o*-XRD) study. X-ray absorption near edge spectroscopy (XANES) data clarified the redox reactions progressed by Ni, Ru, Mn, and O species in the operation range of 1.5–4.2 V; in particular, the observed additional voltage plateau from ~ 3.9 V is supported by the oxidation of lattice oxygen. Different features in the oxygen behavior for both electrodes were highlighted by *operando* differential electrochemical mass spectrometry (*o*-DEMS). The evolution of oxygen was responsible for triggering the oxygen redox reaction to form unpaired electrons in the O 2*p* orbital for the NRM electrode. Based on this result, it is likely that the increased capacity for the V-NRM cathode is closely associated with the pre-existing vacancies in the TM layers, which induce the formation of lone-pair electrons in the O 2*p* orbital in the bulk to support the earlier stage oxidation of oxygen. This behavior eventually results in additional capacity of ~ 21 mAh g^−1^ that corresponds to ~ 0.09 mol Na per formula unit. Herein, we elucidate the role of vacancies in the TM layers for an oxygen redox-derived high-capacity sodium cathode through a combination of experimental and thermodynamic theoretical studies.

## Experimental

### Synthesis

The P2-type Na_0.6_[Ni_0.3_Ru_0.3_Mn_0.4_]O_2_ (NRM) and Na_0.7_[Ni_0.2_V_Ni0.1_Ru_0.3_Mn_0.4_]O_2_ (V-NRM) compounds were produced via a solid-state process. Starting Na_2_CO_3_ (99,5%, Sigma-Aldrich), NiO (99%, Sigma-Aldrich), Mn_2_O_3_ (99%, Sigma-Aldrich), and RuO_2_ (99.9% Alfa Aesar) were mixed with ten ZrO_2_ balls of 5 mm diameter for 1 g of total powder amount by a mixer mill (Retsch, MM400) for 30 min at 30 Hz. The mixture powders were pressed into pellets and calcined in a furnace at 1000 °C for 10 h in dry air (N_2_/O_2_ gas mixture) and then naturally cooled to 200 °C and immediately moved to a dry room.

### Characterization

The as-synthesized resultants were subject to analysis by inductively coupled plasma atomic emission spectroscopy (ICP-AES, OPTIMA 8300, PerkinElmer). The resulting crystal structure was identified using powder X-ray diffraction (XRD; X’Pert, PANalytical) with Cu Kα source (*λ* = 1.5406 Å) in the 2θ range from 10° to 80° with a step size of 0.03°. The data obtained was refined by the FullProf program. The morphology of the as-synthesized material was observed using high-resolution TEM (Hitachi, H-800) and SEM (Hitachi, SU-8010) coupled with energy-dispersive X-ray spectroscopy (EDX). The structural evolution of the material was also monitored by *operando* XRD (*o*-XRD, X’Pert, PANalytical diffractometer) in the 2θ range from 13 to 50° with a step size of 0.03°. Electrical conductivity measurements were carried out using the direct volt–ampere method using the four-point probe technique (CMT-SR1000, AIT). X-ray absorption near edge structure (XANES) spectroscopy measurements were performed at the 8C beamline for Ni and Mn K-edge, at the 10C beamline for the Ru K-edge, and at the 4C beamline for the O K-edge all at the Pohang Accelerator Laboratory (PAL, Pohang, South Korea). The obtained data were analyzed using the Athena software package.

### Electrochemical Testing

The cathodes were fabricated by blending the NRM and V-NRM powders (80 wt%) with Super-P (10 wt%) and polyvinylidene fluoride (PVDF, 10 wt%) in *N*-methyl-2-pyrrolidone (NMP) solution. The obtained material was applied on Al foil using a doctor blade and dried at 120 °C for 12 h. The dried electrodes were punched out with a diameter of 14 ϕ (typically ~ 3 mg cm^−2^). The cathodes were paired with sodium metal disc anodes (16 ϕ) in the presence of 0.5 M NaPF_6_ in propylene carbonate:fluorinated ethylene carbonate at a ratio of 98:2 by volume in R2032 coin cells, of which both electrodes were separated by glass fiber separators (GBR100, Advantec). Typically, electrochemical tests were proceeded by applying a constant current of 26 mA g^−1^ (0.1C) in the voltage range of 1.5 − 4.2 V at 25 °C. Galvanostatic intermittent titration technique (GITT) measurements were progressed at a current pulse of 13 mA g^−1^ with a duration of 1 h and relaxation of 1 h. *Operando* differential electrochemical mass spectrometry (*o*-DEMS) was conducted in a R2032 coin-type cell using stainless steel 50 mesh as the current collector for the cathode. The cells were activated in galvanic mode at a rate of 26 mA g^−1^ (0.1C), for which Ar gas was provided as the carrier gas for both charge and discharge processes at a flow rate of 15 sccm. The gas emissions were measured using a HPR-20 R&D D14 (Hiden Analytical)*.*

### Computational Methods

The NRM and V-NRM structures were modeled using a 4 × 4 × 1 supercell. To identify the favorable configurations of Na_x_[Ni_0.3125_Ru_0.3125_Mn_0.375_]O_2_ (*x* = 0.875 and 0.1875) and Na_x_ [Ni_0.2167_ V_0.098_ Ru_0.3125_ Mn_0.375_] O_2_ (*x* = 0.875 and 0.1875), we generated 3.5 × 10^4^, 9.1 × 10^5^, 2.0 × 10^4^, and 3.8 × 10^5^ structures and computed their coulombic energies (*E*_C_) using the so-called “*Supercell* “ code [36]. The configuration with the lowest *E*_C_ for each case was then fully optimized using DFT calculation. Spin-polarized DFT calculations were performed using the projected augmented wave (PAW) method [37] within the Vienna ab-initio simulation package (VASP). The Perdew–Burke–Ernzerhof (PBE) [38] exchange correlation (XC) functional was applied for the geometry and unit-cell optimization. To compute the electronic structures, we used the Heyd–Scuseria–Ernzerhof (HSE) 06 [39] XC functional with a Hartree–Fock mixing parameter (α) of 0.25 as well as *α* = 0.15 and 0.40. As it is found that the best agreement with experimental data is achieved with *α* = 0.40, the presented electronic structure results in this study are based on this value. A *k*-point mesh grid of (1 × 1 × 1) and energy cutoff of 520 eV were used for all the calculations. Electronic and force convergence criteria of 10^−4^ eV and 2 × 10^−2^ eV Å^−2^, respectively, were applied.

## Results and Discussion

### Material Characterization

**Fig. 1:**
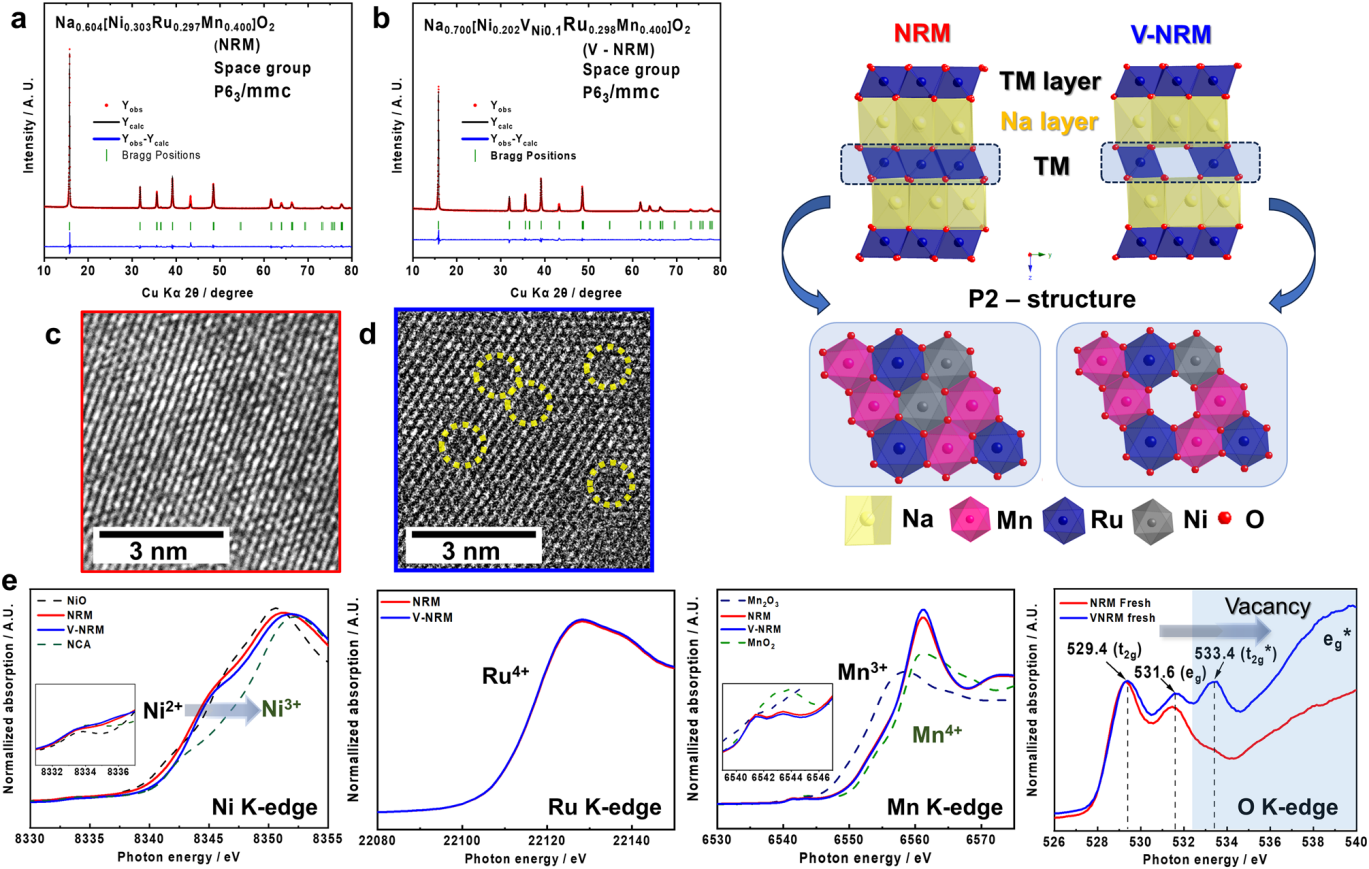
Rietveld refinement of XRD data of **a** NRM and **b** V-NRM and schematic illustration of structures on the right. HR-TEM images of **c** NRM and **d** V-NRM containing vacancy in TM layer. Comparison of **e** XANES results of Ni, Ru, Mn, O between NRM and V-NRM powders

The chemical compositions of the two synthesized compounds were analyzed using ICP-AES (Table S1) and determined to be Na_0.604_[Ni_0.303_Ru_0.297_Mn_0.400_]O_2_ (NRM) and Na_0.702_[Ni_0.202_V_Ni0.1_Ru_0.298_Mn_0.400_]O_2_ (V-NRM). Both synthesized products crystallized into a hexagonal symmetry, *P*6_3_/*mmc*, and there were no impurities in the X-ray diffraction (XRD) patterns for either compound (Fig. 1a, b and Tables S2, S3). The corresponding structures are shown in the top right of Fig. 1. There are differences between the two materials. Specifically, both the *a*-axis parameter was slightly lower for V-NRM (Tables S2 and S3). In V-NRM, the presence of vacancies can result in an increase in the Ni oxidation state for charge compensation, although the Na content was higher to be approximately 0.7 for the V-NRM; while, the Na content was approximately 0.6 for the NRM. The formation of vacancies in the TM layers of V-NRM is apparent from the TEM images (Fig. 1c, d). The emergence of vacancies also affects the oxidation states of the TM elements (Fig. 1e). It is clear from the Ni K-edge XANES spectra that the spectrum associated with V-NRM shifted toward higher photon energy compared with the NRM spectrum. Comparison with Ni^2+^O and Li[Ni^3+^_0.8_Co_0.1_Mn_0.1_]O_2_ (NCA) references indicates that the estimated average oxidation state of Ni is + 2 for NRM and higher than 2 + , partially formation of Ni^3+^, for V-NRM. This finding is in agreement with the Rietveld refinement of XRD data shown in Fig. 1b, Tables S1 and S3, indicating that V-NRM has vacancies in the TM layers of the structure. The formation of Ni^3+^ is related to the formation of vacancies in the TM layers, which satisfies charge balance in the TM layers. The main cause for the decrease in *a*- and *c*- axis parameters is due to the difference in ionic radius between Ni^3+^ (0.56 Å) and Ni^2+^ (0.69 Å). In general, Ni^3+^ provides better electric conductivity than Ni^2+^ in the compound: for instance, 6.5 × 10^–5^ S cm^−1^ for Li[Ni^3+^_0.8_Co_0.1_Mn_0.1_]O_2_ [40], 2.4 × 10^–6^ S cm^−1^ for Li[Ni^2+^_0.5_Mn_0.5_]O_2_ [41], and Li[Ni^2+^_0.5_Mn_0.5_]O_2_ and Li[(Ni^2.25+^_0.5_Mn_0.5_)_0.94_Li_0.06_]O_2_ [42]. For the reason, we measured electric conductivity of NRM and V-NRM materials using four-probe method, of which the presence of the V-NRM sample presents slight improvement in the electric conductivity, ~ 2 × 10^–5^ S cm^−1^ for the NRM and ~ 4 × 10^–5^ S cm^−1^ for the V-NRM. Hence, it is likely that the partial formation of Ni^3+^ is responsible for the slight increase in the electric conductivity. In addition, there were no significant changes in the Ru and Mn K-edge spectra for either sample, indicating both Ru and Mn are stabilized as 4 + . In consideration of the Gibbs free energy for formation (*Δ*G_f_) at 298 K, it is most likely that a variation in the oxidation state of Ni is affected by the differences in the energy among NiO (− 240.6 kJ mol^−1^), RuO_2_ (− 305.0 kJ mol^−1^), and MnO_2_ (− 465.2 kJ mol^−1^) [43–45]. This can be indicative that the Ni^2+^–O bond is weaker than those of Ru^4+^–O and Mn^4+^–O. As a result, it is more favorable to change the oxidation state of Ni rather than Ru and Mn ingredients in the TM layer. Thus, the oxidation state of Ni would increase higher than 2 + to maintain charge balance as the Ni vacancy was formed. Similarly, Dahn’s group reported that the average oxidation state Ni is higher than 2 + in the vacancy Li[Ni_1/6_□_1/6_Mn_2/3_]O_2_ system, where □ denotes vacancy) material containing vacancies [46]. Correspondingly, V-NRM exhibited a slight decrease in both the *a*- and *c*-axis parameters due to the ionic radius of Ni^3+^ (0.56 Å) being smaller than that of Ni^2+^ (0.69 Å).

**Fig. 2:**
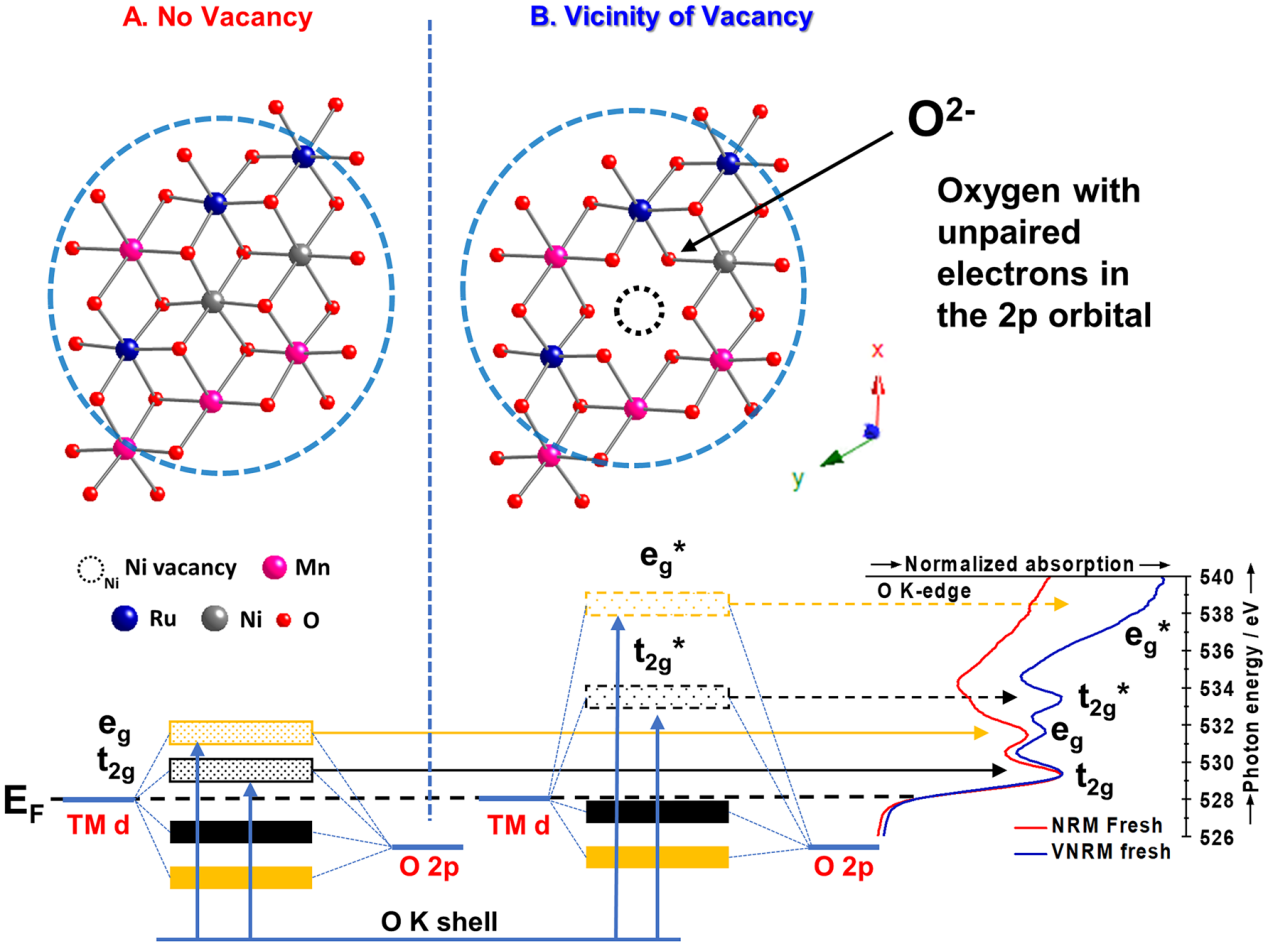
Schematic representation of vacancy influence on O-K edge XANES of V-NRM

Oxygen K-edge XANES revealed the appearance of an additional peak at 533.4 eV for V-NRM due to the presence of vacancies (V_Ni_) in the TM layer. A detailed schematic illustration is included in Fig. 2 to visually represent these findings. For site B, which is a cell near the vacancy, *e*_g_* and *t*_2g_* are farther away from the Fermi level. This situation makes the B site energetically unstable during charging, which is advantageous for sodiation/desodiation. Therefore, *e*_g_* and *t*_2g_* are the first to be filled with electrons. This behavior is reflected in the obtained signal, constituted by the sum of components A and B. The energy difference ΔE becomes broader according to Δτ × ΔE ~ h/2π because the lifetime (Δτ) of electrons excited to *e*_g_* is shorter as they are further away from the Fermi level. Accordingly, the transition peak to *e*_g_* appears broad.

### Electrochemical Performance

**Fig. 3:**
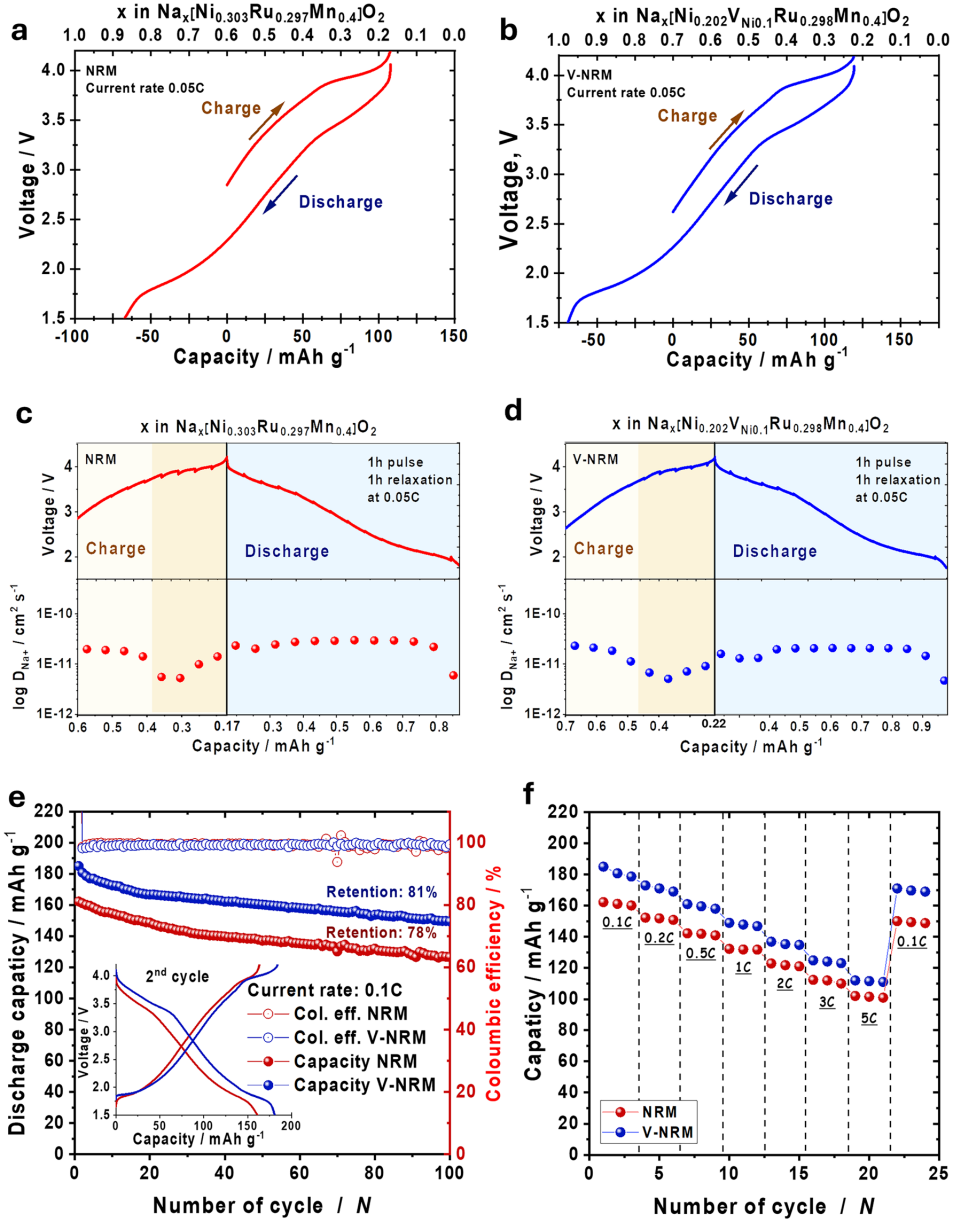
Electrochemical performance comparison of half-cells. Galvanostatic charge–discharge curves of **a** NRM and **b** V-NRM. GITT curves and calculated D_Na+_ diffusion coefficients of **c** NRM and **d** V-NRM. **e** Cyclability comparison and inset of 2nd cycles voltage profiles between NRM and V-NRM. **f** Rate capability comparison between NRM and V-NRM electrodes

The electrochemical activity of NRM and V-NRM was verified in galvanostatic mode by applying 26 mA g^−1^ (0.1C) at 25 °C (Figs. 3a, b and S2e, f). The delivered capacities for the NRM electrode were ~ 101 mAh g^−1^ (~ 0.43 mol Na^+^ extraction) during charging and ~ 163 mAh g^−1^ (~ 0.7 mol Na^+^ insertion) during discharging; whereas, the capacities for the V-NRM electrode were ~ 117 mAh g^−1^ (~ 0.48 mol Na^+^ extraction) for charge and ~ 184 mAh g^−1^ (~ 0.76 mol Na^+^ insertion) for discharge. A gradual rise of the operation voltage was observed for the NRM and V-NRM electrodes until the operation voltage reached 3.9 V (Figs. 3a and S2a, b). However, an additional small plateau like Na^+^/vacancy ordering, corresponding to ~ 0.03 mol of Na^+^ extraction, was observed in the range of 3.75–3.9 V for the V-NRM electrode (Fig. 3b). Similar features were observed in vacancy presenting Na_2_Mn_3_O_7_ [47, 48], Na_0.63_[Mg_0.143_Mn_0.820_V_Mg0.036_]O_2_ [35], and Na_2/3_[Mg_1/9_Mn_7/9_V_Mg1/9_]O_2_ [34]. Moreover, in the voltage range of 3.9–4.2 V, the V-NRM electrode was able to extract a slightly higher amount of Na^+^ (~ 0.02 mol) from the host structure than the NRM electrode. Notably, the increased capacities were ~ 16 mAh g^−1^ on charge and 21 mAh g^−1^ on discharge for the V-NRM electrode compared to those of NRM, although the amount of the active species Ni decreased to 0.2 mol for the V-NRM electrode. Given from the CV data for the first and second cycles (Fig. S2c, d), both NRM and V-NRM electrodes present irreversible behavior for the oxygen redox reaction on the high voltage range, although the extent is not significant. However, such irreversibility is commonly observed in the oxygen redox cathodes [6–15, 21, 22, 30–35, 49], which is most likely associated with the formation of cathodic electrolyte interphase (CEI) layer, composed of NaF or Na-based inorganic compounds [31], on the surface of cathode. This process may consume sodium ingredient to form the CEI layer, such that the capacity obtained at the second discharge would be smaller than that delivered at the first cycle.

The above phenomena imply that the presence of vacancies in the V-NRM electrode can facilitate the overall electrochemical performance in comparison with the vacancy-free NRM one. Thus, the diffusion of Na^+^ was calculated by means of the galvanostatic intermittent titration technique (GITT) (Fig. 3c, d). The diffusion was progressed at a rate of ~ 10^−11^ to ~ 10^−12^ S cm^−1^ for both electrodes in the operation range of 1.5–4.2 V. An abrupt decrease in the diffusion was observed within the range of 0.48 ≤ *x* ≤ 0.22 in Na_*x*_[Ni_0.202_V_Ni0.1_Ru_0.298_Mn_0.400_]O_2_ on charge (Fig. 3d); whereas, the sluggish process appeared rather delayed for the vacancy-free NRM, 0.38 ≤ *x* ≤ 0.17 in Na_*x*_[Ni_0.303_Ru_0.297_Mn_0.400_]O_2_ (Fig. 3c). Such slow kinetics are usually observed when the reaction involves electron transfer through lattice oxygen [19]. Based on the GITT results, it can be inferred that the V-NRM electrode presents a slightly wider range of electrochemical activity associated with the oxidation of lattice oxygen, which may explain the larger capacity compared with that of the vacancy-free NRM electrode. The cycling stability was monitored by applying a current of 0.1C at 25 °C (Fig. 3e). Both electrodes retained reasonable capacity retention: 78% (~ 127 mAh g^−1^) for NRM and 81% (149 mAh g^−1^) for V-NRM after 100 cycles. The rate capability was measured up to 5C (1300 mA g^−1^) (Fig. 3f). The V-NRM electrode had a discharge capacity of 112 mAh g^−1^ at 5C, retaining 61% of the capacity obtained at a rate of 0.1C (Fig. 3f). Although the retention at 5C was similar to 63%, comparing the capacity at 0.1C, the resulting capacity of the NRM electrode was ~ 102 mAh g^−1^ at 5C (Fig. 3f). After the rate test at 5C, both NRM and V-NRM electrodes recovered their capacities to 150.1 and 171.2 mAh g^−1^ at a rate of 0.1C. The above findings demonstrate that the V-NRM structure with the presence of vacancies in the TM layers is beneficial in delivering a higher capacity than the vacancy-free structure, presumably due to the endorsement of the capacity by the lattice oxygen-related reaction at high voltage.

### Energy Storage Mechanism Analysis

**Fig. 4:**
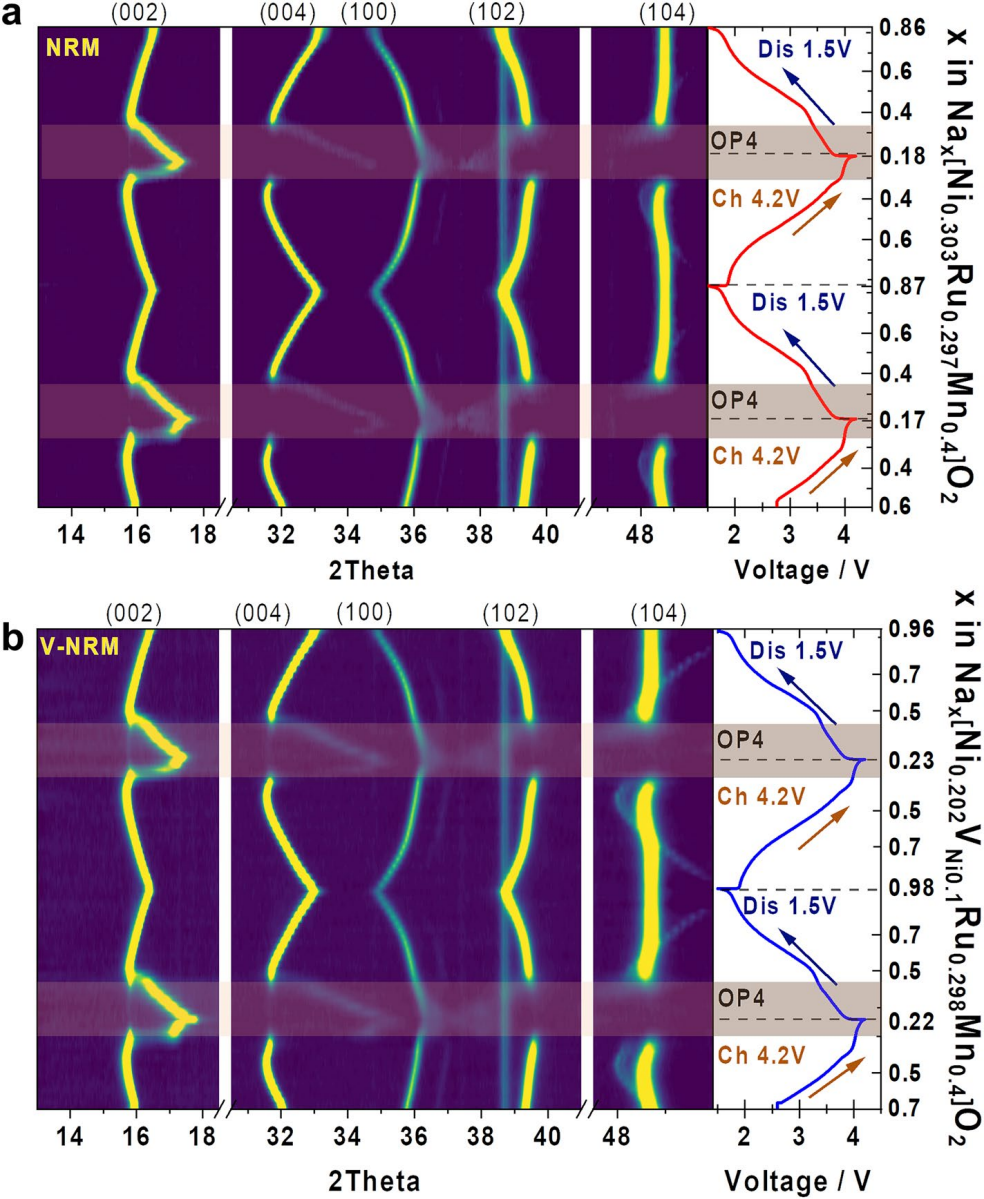
Results of half-cell operando XRD of 2 initial cycles at 0.05C in 2D mapping representing structural evolution of **a** NRM and **b** V-NRM

*Operando* XRD was employed to investigate the structural evolution of NRM and V-NRM electrodes for the first and second cycles (Fig. 4). For the Na_*x*_[Ni_0.303_Ru_0.297_Mn_0.400_]O_2_ electrode (Fig. 4a), extraction of Na^+^ from the host structure induced gradual movements of the (00* l*)_P2_ (*l* = 2 and 4) and (104)_P2_ peaks toward lower angle in the range of 0.6 ≤ *x* ≤ 0.4. Simultaneously, the (100)_P2_ and (102)_P2_ Bragg peaks shifted toward a higher angle. As the Na^+^ extraction continued in the range of 0.4 ≤ *x* ≤ 0.3 in Na_*x*_[Ni_0.303_Ru_0.297_Mn_0.400_]O_2_ (~ 3.7 to ~ 3.95 V), the observed Bragg peaks moved toward higher angle within the P2 structure framework. This range 0.4 ≤ *x* ≤ 0.3 in Na_*x*_[Ni_0.303_Ru_0.297_Mn_0.400_]O_2_ (~ 3.7 to ~ 3.95 V) shows a monotonous rise of operation voltage. From 3.95 to 4.2 V, there is a flat plateau, corresponding to the 0.25 ≤ *x* ≤ 0.17 range in Na_*x*_[Ni_0.303_Ru_0.297_Mn_0.400_]O_2_. In this range, the original P2 phase has vanished, and a new OP4 phase emerges, resulting in the composition Na_0.17_[Ni_0.303_Ru_0.297_Mn_0.400_]O_2_ at the end of the charging process. The reversible behavior is observed throughout the discharge process. The OP4 phase is retained to *x* = 0.35 in Na_*x*_[Ni_0.303_Ru_0.297_Mn_0.400_]O_2_ (~ 3.4 V), after which the phase transforms to P2 and is predominant to the end of discharge, resulting in Na_0.87_[Ni_0.303_Ru_0.297_Mn_0.400_]O_2_. This phase transition from P2 to OP4 is further progressed during the second charge, and vice versa on the second discharge.

For the V-NRM electrode (Fig. 4b), the associated phase transition is the same as for the NRM electrode, namely, P2 → OP4 on charge and vice versa on discharge. After the small plateau mentioned in Fig. 3b, which was observed in the range of 3.75–3.9 V (0.43 ≤ *x* ≤ 0.40), the P2 framework of V-NRM undergoes a transformation to the OP4 phase at a high-voltage plateau within the range of 0.40 ≤ *x* ≤ 0.22. During discharge, the regime of the OP4 phase was maintained in the range of 0.22 ≤ *x* ≤ 0.43 until the voltage reached 3.3 V. After this point, the recovered P2 structure became dominant and persisted throughout the Na^+^ insertion process until the end of discharge, resulting in a composition of Na_0.98_[Ni_0.202_V_Ni0.10_Ru_0.298_Mn_0.400_]O_2_. The above findings demonstrate that the increased capacity can be attributed to the widened dominance of the OP4 phase in the high-voltage region.

**Fig. 5:**
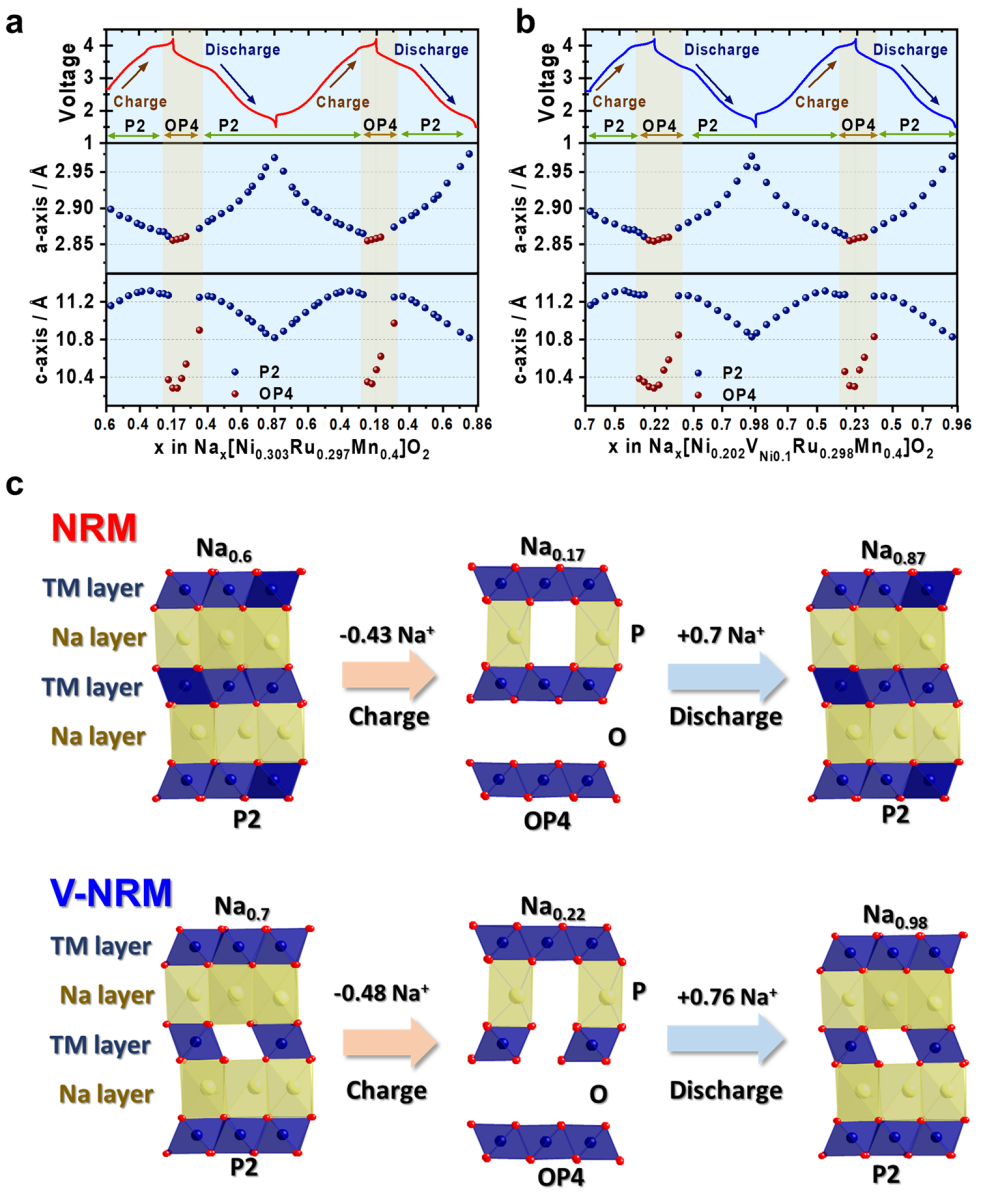
Variations in lattice parameters (*a*- and *c*-axis) during operando XRD analysis of **a** NRM and **b** V-NRM. Panel **c** provides a schematic illustration of the corresponding structural changes observe

The lattice parameters were calculated using the least squares method obtained from *o*-XRD patterns (Fig. 5a, b). The tendency in the variation of the *a*- and *c*-axes was similar during Na^+^ de-/intercalation for both the NRM and V-NRM electrodes. The variation in the *a*-axis showed a gradual decrease with a sharper slope within the P2 phase than in the OP4 region. Both electrodes underwent a typical change in the *c*-axis variation in the P2-phase region. Namely, an increase in the *c*-axis value is due to the increased oxygen–oxygen electrostatic repulsion in the interlayer during desodiation. However, as the desodiation progresses, the covalent character becomes more dominant over the electrostatic one in the interlayer of the P2 phase, leading to a decrease in the *c*-axis parameter. Further, the OP4 phase emerged in the highly desodiated state, which caused an abrupt change in the *c*-axis parameters for both the NRM and V-NRM electrodes. The large change in the *c*-axis is caused by the formation of an ordered OP4 phase with the alternating octahedral (O) (highly Na^+^ depleted O-type) and prismatic (P) (Na-containing P-type) layers. Similar phenomena are typically observed in P2-type cathode materials [50–53]. However, the change in the *c*-axis did not affect the cyclability of the materials, as observed in Fig. 3e. These lattice parameters recovered during sodiation to the original values at the original Na contents Na_0.6_[Ni_0.3_Ru_0.3_Mn_0.4_]O_2_ (NRM) and Na_0.7_[Ni_0.2_V_Ni0.1_Ru_0.3_Mn_0.4_]O_2_. Further sodiation led to an increase in the *a*-axis value with progressive reduction of TM elements and a decrease in the *c*-axis value due to the decreased oxygen–oxygen electrostatic repulsion in the interlayer. The tendency was repeated in the second cycle. The associated structural changes for both NRM and V-NRM are schematically illustrated in Fig. 5c.

The variation of lattice parameters of the V-NRM presented the similar tendency to the NRM electrode during de/sodiation. There are two main contributors to the enhanced cycling stability: (i) first is the presence of Ni^3+^ in the structure of V-NRM that can provide improved electric conductivity than Ni^2+^, associated with the overlapping density of states between the O 2*p* and Ni 3*d* (Ni^4+^/Ni^3+^) orbitals. This increase in the conductivity enhances the cycling stability to have more revisable migration Na^+^ into/out of the host structure. (ii) Second is that the presence of vacancies can suppress the stacking faults in the Na layer which are unfavorable for the structure stability [49, 54]. The oxidation of oxygen occurs more favorably in presence of vacancy in the TM layers, accompanied by the charge compensation with Na^+^ ions. The existing covalent character by Ru supports the reversible behavior. Therefore, it is likely that the formation of TM vacancies is responsible for the formation of partial Ni^3+^, which spontaneously triggers participation of Na^+^ ions for charge compensation process that improves the capacity. And the existing covalency Ru–O bond in the TM layer endows the structural stability over cycling.

**Fig. 6:**
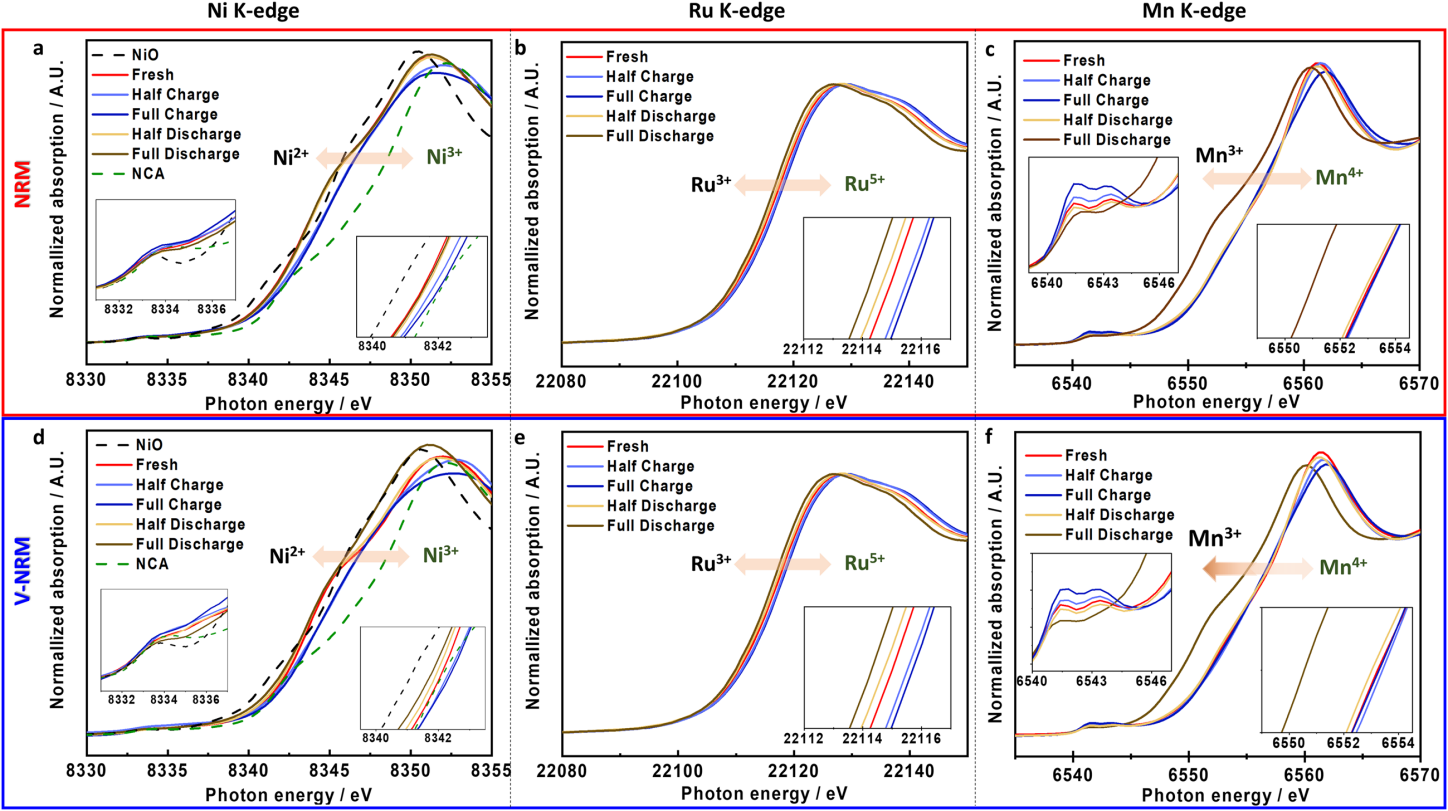
XANES spectra comparison between **a**-**c** NRM and **d**-**f** V-NRM of Ni-K edge (**a, d**), Ru-K edge (**b, e**), and Mn-K edge (**c, f**)

As discussed in Fig. 3, the V-NRM electrode delivered higher capacity than the NRM electrode, approximately 16 mAh g^−1^ on charge and 21 mAh g^−1^ on discharge, although the Ni content was decreased to 0.2 mol for V-NRM. Hence, both electrodes were subject to investigation using XANES to follow the charge compensation during de/sodiation (Fig. 6). For the NRM electrode (Fig. 6a), there was a positive shift of the photon energy to the half-charge point (Na_0.*39*_[Ni_0.303_Ru_0.297_Mn_0.400_]O_2_), indicating that Ni^2+^ was oxidized to Ni^3+^. In addition, Ni was inactive in the photon energy by the end of charge, reaching Na_0.*17*_[Ni_0.303_Ru_0.297_Mn_0.400_]O_2_. In addition, the Ru K-edge spectra shifted to higher proton energy via the desodiation to the half-charge point, and there was an additional small shift in the photon energy upon further desodiation to Na_0.*17*_[Ni_0.303_Ru_0.297_Mn_0.400_]O_2_ (Fig. 6b). As expected, Mn was not active in the entire range on charge, as the initial Mn^4+^ cannot be further oxidized beyond its state (Fig. 6c). During sodiation, it is evident that Ni is reduced to the original state of Ni^2+^ at the half discharge point (Na_0.*52*_[Ni_0.303_Ru_0.297_Mn_0.400_]O_2_), below which Ni was inactive to the end of discharge (Na_0.*87*_[Ni_0.303_Ru_0.297_Mn_0.400_]O_2_) (Fig. 6a). It is also notable that the main shift of the Ru spectra was perceived to the half discharge point, and an additional slight shift toward Ru^3+^ was also visible to the end of discharge (Fig. 6b). Mn was inactive to the half discharge point, below which its participation in the reduction process was active to 1.5 V, indicating reduction toward Mn^3+^ (Fig. 6c).

In a similar manner, the V-NRM electrode is activated during de/sodiation (Fig. 6d–f). Evidently, Ni is active to the half-charge point (Na_0.46_[Ni_0.202_V_Ni0.1_Ru_0.298_Mn_0.400_]O_2_) (Fig. 6d), and the V-NRM electrode exhibited a more shift of the photon energy compared with the NRM electrode (Fig. 6a). There were no large differences in the activities of Ru and Mn on charge (Fig. 6e, f) compared to those of the NRM electrode. That is, Ru was oxidized toward 5 + but Mn remained as inactive Mn^4+^ upon desodiation. During sodiation, the reduction of Ni to the initial state was evident at the half discharge point (Na_*0.59*_[Ni_0.202_V_Ni0.1_Ru_0.298_Mn_0.400_]O_2_), below which a slight additional shift of Ni toward Ni^2+^ was observed by the end of discharge (Na_0.98_[Ni_0.202_V_Ni0.1_Ru_0.298_Mn_0.400_]O_2_) in Fig. 6d. Similar to the Ru activity in the NRM electrode, the shift of Ru was evident toward Ru^3+^ (Fig. 6e). Mn did not show the electrochemical activity up to the half discharge point; however, the reduction progressed toward Mn^3+^ by the end of discharge, exhibiting a more shift relative to that of NRM electrode. These movements of TM elements provide important insight on the charge compensation process. The presence of vacancies in the TM layers originally induces an increase in the Ni oxidation state, such that the partially formed Ni^3+^, which is more electroconductive than Ni^2+^ due to the overlapping density of states between the O 2*p* and Ni *3d* (Ni^4+^/Ni^3+^) orbitals, provides a favorable activity.

**Fig. 7:**
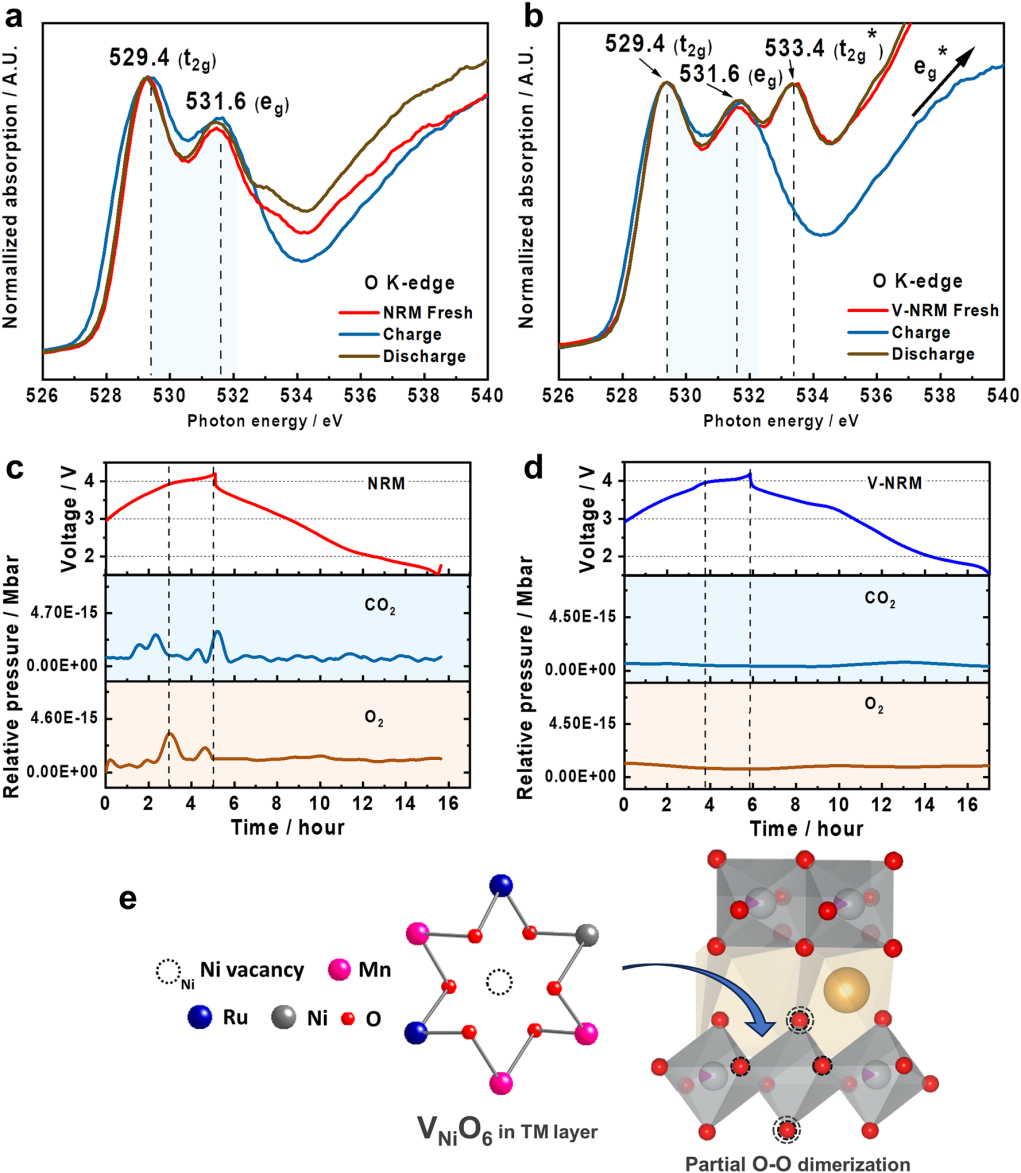
O-K edge XANES spectra of **a** NRM and **b** V-NRM. *Operando* differential electrochemical mass spectrometry (DEMS) of the rate of O_2_ and CO_2_ evolution of **c** NRM and **d** V-NRM during the initial cycle. **e** Schematic structure of V_Ni_ in TM layers

According to the XANES data, the TM redox pairs were primarily active below 4 V during the charging process, which suggests that the capacity obtained at voltages above 4 V could be attributed to the progress of oxygen-related activity. Therefore, the O K-edge XANES spectra were measured in total-fluorescence-yield (TFY) mode to identify the oxygen behavior for both electrodes (Fig. 7a, b). Two pre-edge peaks emerged at ~ 529.4 (*t*_2g_) and 531.6 eV (*e*_g_) due to the hybridized state of the TM 3*d/4d* – O 2*p* orbitals. The normalized intensity of the peak at 531.6 eV increased, indicating the formation of oxidized species in the *e*_g_ orbital and the formation of lone-pair electrons for both NRM and V-NRM in the absorption energy spectra. It is interesting to note that the relative intensity for 531.6 eV (*e*_g_) was slightly higher for V-NRM even in its fresh state (Fig. 7b) than for NRM (Fig. 7a). Additionally, peaks related to *e*_g_* and *t*_2g_* in V-NRM disappeared during charging and were restored after discharge, suggesting that the fresh V-NRM material already contains the oxidized lattice oxygen with lone-pair electrons in the O 2*p* orbital due to the presence of vacancies (V_Ni_) in the TM layers. This phenomenon could affect the delivery of higher capacity contributed by the reaction of oxygen redox over 4 V. After discharge, the intensity of the 531.6 eV peak (*e*_g_) was recovered to that of the fresh state for both samples, indicating the reversibility of the oxygen redox process. Since more lone-pair electrons in the lattice oxygen participate in the redox process for the V-NRM, the sluggish electron transfer by the oxygen redox is likely to affect slightly inferior rate capability to the NRM electrode at high rates although absolute value of discharge capacity is higher for the V-NRM electrode. Additionally, the results of the O-K edge XANES were supported by XPS (Fig. S4), which showed similar differences in the results. Based on the XANES measurements, we can conclude that the high-voltage region was mainly responsible for the activity of oxygen, Ni^3+^/Ni^2+^ and Ru^5+^/Ru^3+^ redox pairs; whereas, the Mn^4+^/Mn^3+^ redox pair was active below 2.4 V.

It is noteworthy that, as mentioned in the introduction, one of the criteria for the oxygen redox is either the migration of elements from TM to Na layers or the evolution of lattice oxygen. Each case leads to the formation of lone-pair electrons in the O 2*p* orbital. The NRM electrode also exhibited the oxygen redox as shown in Fig. 7a, showing an overlapping between *t*_2g_ and *e*_g_ orbitals after charging to 4.2 V. In this case the oxygen redox is progressed after evolution of lattice oxygen (Fig. 7c). The oxygen release also results in the generation of lone-pair electrons in the O 2*p* orbital to induce the oxidation of oxygen. Additionally, below 4 V we observe CO_2_ evolution in *o*-DEMS was due to the surface sodium carbonate residue, Na_2_CO_3_, and above 4 V, CO_2_ resulting from the oxidative decomposition of the electrolyte. Strikingly, the V-NRM did not show such evolution of lattice oxygen during de/sodiation, but a similar tendency on the CO_2_ release was observed below 4 V for V-NRM (Fig. 7d). This clearly indicates that the introduction of vacancies into the TM layer allows a more adaptable structure for de-/sodiation, improving the structural integrity owing to the no evolution of lattice oxygen from the structure. As a result, the V-NRM delivered higher capacity (approximately 23 mAh g^−1^) than the original NRM electrode. The V-NRM electrode experienced variation in the 531.6 eV (*e*_g_) spectrum, which is attributed to the oxidation of lattice oxygen triggered by the presence of vacancies (V_Ni_) in the TM layers (Fig. 7b). This finding indicates that the oxygen in the V_Ni_O_6_ octahedra are not bonded with the Ni element and spontaneously produces the unpaired electrons in the O 2*p* orbital (Fig. 7e). Moreover, no oxygen was evolved from the structure; however, the oxidation of oxygen spontaneously progresses, accompanied by charge compensation with Na^+^ extraction on charge and vice versa on discharge. From the portion of the oxygen participation and XANES spectra, oxygen would behave like dimerized oxygen, (O_2_)^n−^ (*n* < 2) from O^2−^ in the oxide lattice.

### DFT Calculations

**Fig. 8:**
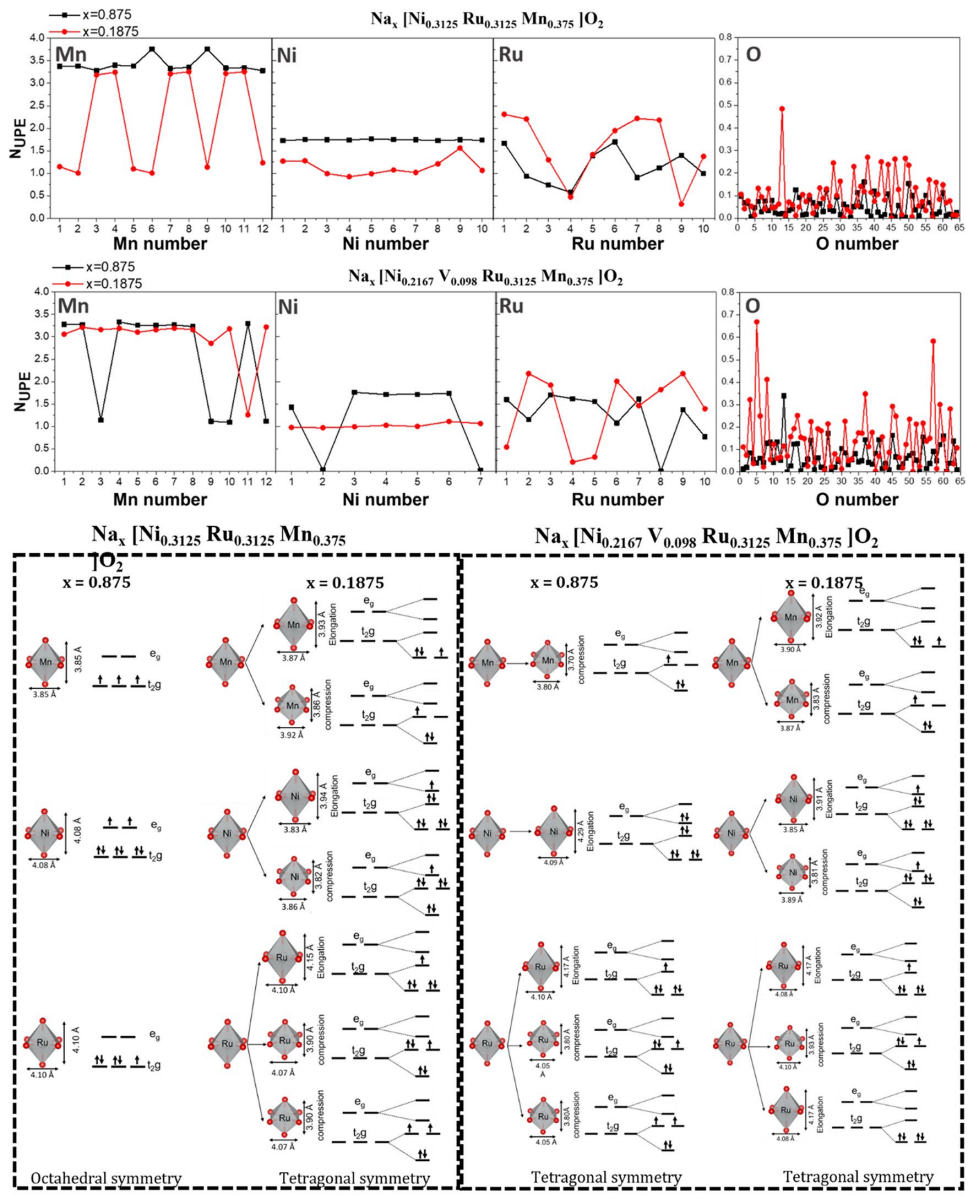
Calculated number of unpaired electrons (N_UPE_) and possible orbital splitting for active transition metal ions in Na_x_[Ni_0.3125_Ru_0.3125_Mn_0.375_]O_2_ and Na_x_ [Ni_0.2167_V_0.098_Ru_0.3125_Mn_0.375_] O_2_ in discharged (*x* = 0.875) and charged (*x* = 0.1875) states

**Fig. 9:**
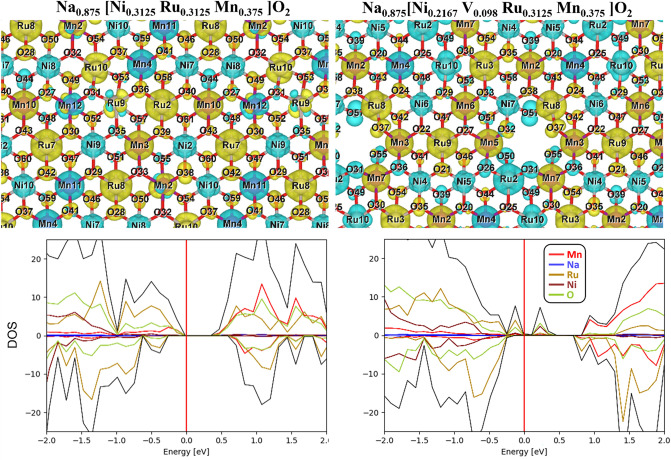
Calculated spin density difference of one of the O-TM-O layers and projected total density of states on elements for NRM and V-NRM at high sodium concentration (x= 0.875)

The computed atomistic structures of NRM and V-NRM using DFT-PBE show that the introduction of 3% Ni vacancies into NRM results in the migration of a small fraction of Na ions (3.5%) into the TM sites in V-NRM as shown in Fig. S5. This behavior can stem from (i) a strong Na–Na repulsion, which can be reduced by Na migration from the Na to TM plane and/or (ii) instability of TM vacant sites at *x*_Na_ = 0.875. However, after charging, when a large concentration of unoccupied Na sites in the Na plane is available, Na cations leave the TM sites. To understand the charge/discharge process, the redox mechanism of TM and oxygen was studied by computing the number of unpaired electrons N_UPE_ (i.e., the magnetic moment). Figure 8 shows that for the vacancy-free system (NRM) at high Na concentration (Na_0.875_[Ni_0.3125_Ru_0.3125_Mn_0.375_]O_2_), the calculated average value of N_UPE_ (${\overline{N} }_{\text{UPE}}$) for the Mn cation is $=$ 3.41, indicating a charge of 3.59 + . For desodiated NRM with *x*_Na_ = 0.1875, half of the Mn cations have a ${\overline{N} }_{\text{UPE}}$ value of 3.23; whereas, the other half have a value of 1.10. The former ${\overline{N} }_{\text{UPE}}$ can be assigned to a charge state of 3.77 + as per the typical octahedral splitting of the *d* orbitals into *t*_2g_ and *e*_g_ orbitals (Fig. 8). However, for the Mn cations with ${\overline{N} }_{\text{UPE}}$=1.10, we generally find that neighboring oxygens experience a relatively large oxidation, which might have an effect on the energy states of *d* orbitals. The oxidation of oxygen anions, which will be discussed later (Fig. 9), cause tetragonal elongation or compression of octahedral oxygen cages (Fig. 8). This will result in the formation of tetragonal MnO_6_, along with octahedral MnO_6_. Two corresponding splitting schemes were used to describe the *d* orbitals of Mn cations in tetragonal MnO_6_ showing that the ${\overline{N} }_{\text{UPE}}$ value of 1.10 (Fig. 8) can be assigned to a charge state of 3.90 + . The average charge state of Mn after desodiation is, therefore, estimated to be (3.77 + 3.90)/2 $\approx$ 3.84 + . This finding indicates that Mn cations experience an oxidation of 3.59 + to 3.84 + for *x*_Na_ = $0.875\to 0.1875$. Ni cations, however, experience a relatively large oxidation from 2.26 + to 2.86 + , which is estimated from ${\overline{N} }_{\text{UPE}}$ values of 1.74 and 1.14, respectively, considering the normal octahedral symmetry (octahedral NiO_6_) for sodiated case and tetragonal symmetry (tetragonal NiO_6_) for desodiated case (Fig. 8). This result confirms our XANES measurement (Fig. 6a) showing that Ni^2+^ is oxidized to Ni^3+^ after desodiation. Considering the octahedral symmetry, the computed ${\overline{N} }_{\text{UPE}}$ value of 1.14 for Ru cations in RuO_6_ octahedra in Na_0.875_[Ni_0.3125_Ru_0.3125_Mn_0.375_]O_2_ shows a charge state of 3.14 + . In the desodiated structure, namely Na_0.1875_[Ni_0.3125_Ru_0.3125_Mn_0.375_]O_2_, the computed Ru–O bond lengths also show that all the octahedral oxygen cages with Ru cations undergo tetragonal distortion, and hence have a tetragonal symmetry, as shown in Fig. 8. 50% of tetragonal RuO_6_ have Ru cations with a ${\overline{N} }_{\text{UPE}}$ value of 2.17 possessing an average charge state of 3.83 + , 30% of tetragonal RuO_6_ have Ru cations with ${\overline{N} }_{\text{UPE}}$ =1.36, having an average charge state of 4.64 + , and 20% tetragonal RuO_6_ have Ru with ${\overline{N} }_{\text{UPE}}$ =0.39, and hence have an average charge state of 3.61 + . The desodiation-induced oxidation of Ru depends on its nearest neighbor (NN): when the NN of Ru are Mn and Ni, Ru cations experience relatively large oxidations (for example Ru8 in Fig. 9: 3.14+$\to$ 3.81 +). However, when Ru cations are NN, the oxidation of one of them is lowered (for example Ru10 in Fig. 9: 3.14+$\to$ 3.37 +), which is probably enforced by avoiding a strong electrostatic repulsion between neighboring Ru cations with high oxidation states. The average charge on Ru cations in Na_0.1875_[Ni_0.3125_Ru_0.3125_Mn_0.375_]O_2_ (from Fig. 8) is (0.5×3.83 + 0.3×4.64 + 0.2×4.39)/3 = 4.19 + , which lies at the low end of the range determined by our experimental data (between 4 and 5) (Fig. 6b). A strong overlap of *d* orbitals of Ru cations with *p* orbitals of O anions (see the computed DOS in Fig. 9) and oxidation of O might explain the low charge state on Ru. Figure 9 indicates that each Ru ion has some neighboring oxygen anions that are partially oxidized (e.g. O46 and O50 close to Ru8). The calculated *N*_UPE_ values in Fig. 8 indicate that O undergoes oxidation from 1.95 $-$ (${\overline{N} }_{\text{UPE}}$=0.05) to 1.90 $-$ (${\overline{N} }_{\text{UPE}}$=0.10) for *x*_Na_ = 0.875 $\to$ 0.1875.

For the discharged case of the cathode material with Ni vacancies, Na_0.875_[Ni_0.2167_V_0.098_Ru_0.3125_Mn_0.375_]O_2_, octahedral oxygen cages are generally distorted in the presence of Ni vacancies. Considering the octahedral and tetragonal symmetry, we find that 67% and 33% of Mn cations have a charge state of 3.73 + (Octahedral MnO_6_: ${\overline{N} }_{\text{UPE}}$(Mn) = 3.27) and 3.88 + (tetragonal MnO_6_ ${\overline{N} }_{\text{UPE}}$(Mn) = 1.12), respectively. The average charge of Mn is, therefore, computed to be 3.78 + . After desodiation (Na_0.1875_[Ni_0.2167_V_0.098_Ru_0.3125_Mn_0.375_]O_2_), the average charge state of Mn is 3.86 + (92% Octahedral MnO_6_: ${\overline{N} }_{\text{UPE}}$(Mn) = 3.13 and 8% tetragonal MnO_6_: ${\overline{N} }_{\text{UPE}}$(Mn) = 1.26), showing a slight desodiation-induced oxidation of 3.78+ $\to$ 3.86 + . Considering octahedral and tetragonal symmetry, the computed average charge on Ni before and after desodiation is 2.22 + (71% octahedral NiO_6_: ${\overline{N} }_{\text{UPE}}$(Ni) = 1.67, 29% tetragonal NiO_6_: ${\overline{N} }_{\text{UPE}}$(Ni) = 0.02) and 2.98 + (tetragonal NiO_6_ ${\overline{N} }_{\text{UPE}}$(Ni) = 1.02), respectively (Fig. 8). This result confirms our experimental data (Fig. 6d), demonstrating an increase in the oxidation state of Ni toward Ni^3+^ to maintain charge balance when the Ni vacancy is formed. The average charge state on Ru before desodiation is estimated to be 3.45 + (50% tetragonal RuO_6_: ${\overline{N} }_{\text{UPE}}$(Ru) = 1.62, 40% tetragonal RuO_6_: ${\overline{N} }_{\text{UPE}}$(Ru) = 1.09, 10% tetragonal RuO_6_: ${\overline{N} }_{\text{UPE}}$(Ru) = 0.01), which increases to 4.21 + upon desodiation (*x*_Na_ = 0.875 $\to$ 0.1875), being very similar to Ru in the NRM system. Similar to the NRM case, a strong overlap between *d* orbitals of Ru cations and *p* orbitals of O anions is observed in the computed DOS plot of Fig. 9. The peak O-*p* orbitals and their overlap with the Ru-*d* orbitals are closer to the Fermi level for V-NRM, indicating a larger tendency for oxidation of O in this system compared to NRM. The computed higher values of N_UPE_ (Fig. 8) as well as the larger spin density difference on O (Fig. 9) for the desodiated V-NRM confirms the DOS result. The value of ${\overline{N} }_{\text{UPE}}$ in V-NRM changes from 0.06 → 0.14 upon desodiation, showing an oxidation of 1.94 $-$→ 1.86 $-$, which is 0.03 (per O) larger than that in NRM. In particular, there are eight O anions with ${\overline{N} }_{\text{UPE}}$ equal or larger than 0.30 for the V-NRM case, while only one O for the NRM case (Fig. 8). Five out of eight largely oxidized O in the former case are located next to the Ni vacant sites. This result shows that although the oxidation of Ru is larger in NRM, but oxidation of O and Ni is higher in V-NRM. We might even have a larger oxidation of O than what predicted from DFT calculation for the V-NRM case due to the applied computational approximations (e.g., the Perdew–Burke–Ernzerhof exchange correlation functional and atomic projection). The above findings indicate the formation of additional lone-pair electrons in the O 2*p* orbital enables oxygen redox, leading to a widened dominance of the OP4 phase by suppressing the release of O_2_. Therefore, the vacancies in the TM layers play a pivotal role to result in enhanced electrode performance for oxygen redox-derived high-capacity cathode materials, which provides insights for design of high-capacity active materials.

## Conclusions

In summary, the introduction of vacancies into the TM layer of the NRM cathode material with a hexagonal P2 structure allows for a more adaptable structure for de-/sodiation, providing more space for sodium. The resulting vacancy-containing material, V-NRM, exhibits a higher capacity (approximately 23 mAh g^−1^ more) than the original NRM and retains a moderate capacity of 81% after 100 cycles at 0.1C. The vacancies also result in the generation of additional lone-pair electrons in the oxygen 2*p* orbital, facilitating a greater utilization of oxygen redox and expanding the dominance of the OP4 phase. Importantly, the introduction of vacancies induces oxygen redox without the formation of O_2_ and CO_2_ gases, as revealed by operando DEMS. Overall, this study suggests that deliberately introducing vacancies in the TM layer can enhance the capacity, stability, and electrochemical properties of hexagonal P2 structured cathode materials, offering new possibilities for the development of advanced cathode materials for sodium-ion batteries.

## Supplementary Information

Below is the link to the electronic supplementary material.

Supplementary file1 (PDF 986 KB)
