# Correction: Impact of Transition Metal Layer Vacancy on the Structure and Performance of P2 Type Layered Sodium Cathode Material

**DOI:** 10.1007/s40820-024-01492-4

**Published:** 2024-08-30

**Authors:** Orynbay Zhanadilov, Sourav Baiju, Natalia Voronina, Jun Ho Yu, A-Yeon Kim, Hun-Gi Jung, Kyuwook Ihm, Olivier Guillon, Payam Kaghazchi, Seung-Taek Myung

**Affiliations:** 1 Department of Nanotechnology and Advanced Materials Engineering and Sejong Battery Institute, Hybrid Materials Research Center, Sejong University, 98 Gunja-Dong, Gwangjin-Gu, Seoul, 05006 South Korea; 2 Institute of Energy and Climate Research-Materials Synthesis and Processing (IEK-1), Forschungszentrum Jülich GmbH, 52425 Jülich, Germany; 3 Center for Energy Storage Research, Korea Institute of Science and Technology, Seoul, 02792 South Korea; 4 KIST-SKKU Carbon-Neutral Research Center, Sungkyunkwan University, Suwon, 16419 South Korea; 5 Department of Energy Science, Sungkyunkwan University, Suwon, 16419 South Korea; 6 Pohang Accelerator Laboratory, 80 Jigokro-127-Beongil, Nam-Gu, Pohang, Gyeongbuk 37673 South Korea; 7 MESA+ Institute for Nanotechnology, University of Twente, 7500 AE Enschede, The Netherlands

**Correction to: Nano-Micro Lett. (2024) 16:239** 10.1007/s40820-024-01439-9

Following publication of the original article [1], the authors reported that the author Hun-Gi Jung should be affiliated as 3, 4 and 5 instead of 4 and 5. The author's name “A.-Yeon Kim” needed to be updated to “A-Yeon Kim”, removing the period.

The correct author’s name and affiliation have been provided in this Correction. The original article [1] has been corrected.
