# Supplementary material for: Impact of Transition Metal Layer Vacancy on the Structure and Performance of P2 Type Layered Sodium Cathode Material
Source: Nanomicro Lett. 2024 Jul 8;16:239. doi: 10.1007/s40820-024-01439-9 (PMC11231127; doi:10.1007/s40820-024-01439-9)
Supplement: Supplementary file 1 — Supplementary file1 (PDF 986 KB) [file 40820_2024_1439_MOESM1_ESM.pdf]

Supporting Information for

Impact of Transition Metal Layer Vacancy on the Structure and Performance of P2 Type Layered Sodium Cathode Material

Orynbay Zhanadilov[1,†], Sourav Baiju[2,†], Natalia Voronina[1], Jun Ho Yu[1], A-Yeon Kim[3], Hun-Gi Jung[4,5], Kyuwook Ihm[6], Olivier Guillon[2], Payam Kaghazchi[2,7,*], Seung-Taek Myung[1,*]

[1] Hybrid Materials Research Center, Department of Nanotechnology and Advanced Materials Engineering & Sejong Battery Institute, Sejong University, 98 Gunja-dong, Gwangjin-gu, Seoul 05006, South Korea

[2] Institute of Energy and Climate Research-Materials Synthesis and Processing (IEK-1), Forschungszentrum Jülich GmbH, 52425 Jülich, Germany

[3] Center for Energy Storage Research Korea Institute of Science and Technology, Seoul 02792, South Korea

[4] KIST-SKKU Carbon-Neutral Research Center, Sungkyunkwan University, Suwon 16419, South Korea

[5] Department of Energy Science, Sungkyunkwan University, Suwon 16419, South Korea

[6] Pohang Accelerator Laboratory, 80 Jigokro-127-beongil, Nam-gu, Pohang, Gyeongbuk 37673, South Korea

[7] MESA+ Institute for Nanotechnology, University of Twente, 7500 AE Enschede, the Netherlands

†Orynbay Zhanadilov and Sourav Baiju contributed equally to this work.

*Corresponding authors. E-mail: p.kaghazchi@fz-juelich.de (Payam Kaghazchi); smyung@sejong.ac.kr (Seung-Taek Myung)

Supplementary Tables and Figures

Table S1 ICP-AES results comparison of pristine NRM and V-NRM powders

Theoretical chemical formula	Na	Ni	Mn	Ru
$Na_{0.604}[Ni_{0.303}Ru_{0.297}Mn_{0.400}]O_2$	0.604	0.303	0.400	0.297
$Na_{0.702}[Ni_{0.202}V_{Ni0.1}Ru_{0.298}Mn_{0.400}]O_2$	0.702	0.202	0.400	0.298

Table S2 Rietveld refinement results of the XRD data for $Na_{0.604}[Ni_{0.303}Ru_{0.297}Mn_{0.400}]O_2$ (NRM)

Atom	*x*	*y*	*z*	*g*	B_{iso}/Å^2	Site
Na_e	0.66667	0.33333	0.25	0.362(1)	3.3	2*d*
Na_f	0	0	0.25	0.23(6)	3.0	2*b*
O	0.33333	0.66667	0.0882(1)	1.0	0.1	4*f*
Ni	0	0	0	0.303(2)	0.5	2*a*
Mn	0	0	0	0.4(2)	0.5	2*a*
Ru	0	0	0	0.297(2)	0.5	2*a*

Hexagonal/Space group: $P6_3/mmc$
Cell parameter: a=b=2.9128(4)Å, c=11.2554(1)Å, V=82.57(6)Å^3, α=β=90°, γ=120°
R_{wp}% = 5.02%

Table S3 Rietveld refinement results of the XRD data for $Na_{0.702}[Ni_{0.202}V_{Ni0.1}Ru_{0.298}Mn_{0.400}]O_2$ (V-NRM)

Atom	*x*	*y*	*z*	*g*	B_{iso}/Å^2	Site
Na_e	0.66667	0.33333	0.25	0.462(3)	3.3	2*d*
Na_f	0	0	0.25	0.24(2)	3.0	2*b*
O	0.33333	0.66667	0.0957(5)	1.0	0.9	4*f*
Ni	0	0	0	0.202(1)	0.5	2*a*
Mn	0	0	0	0.4(1)	0.5	2*a*
Ru	0	0	0	0.298(1)	0.5	2*a*

Hexagonal / Space group: $P6_3/mmc$
Cell parameter: a=b=2.9105(9)Å, c=11.2075(8)Å, V=82.35(2)Å^3, α=β=90°, γ=120°
R_{wp}% = 4.12%

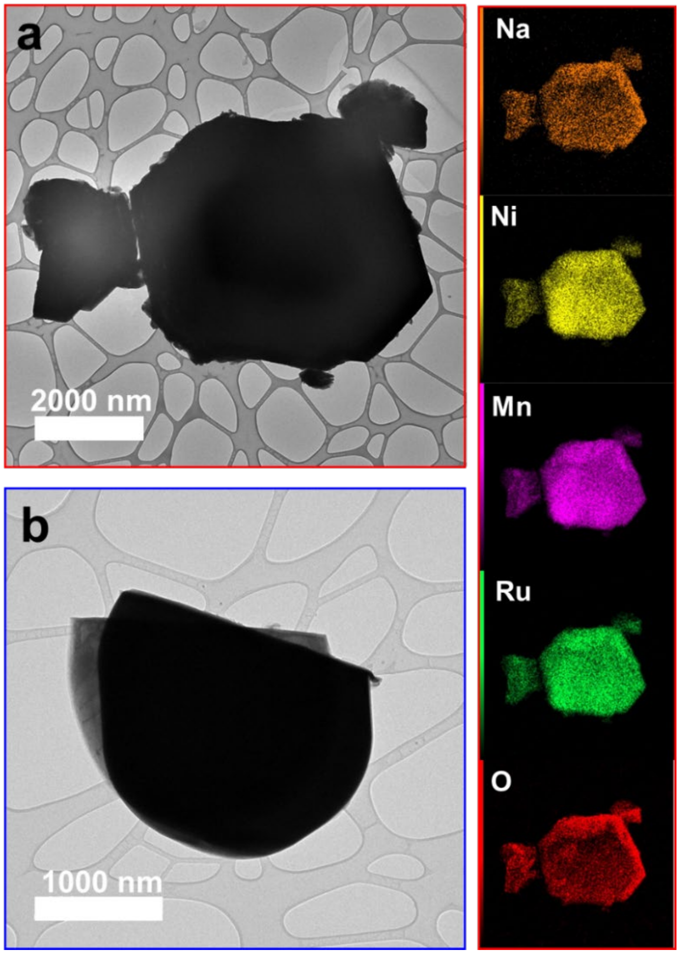

Fig. S1 TEM image and TEM-EDS elemental mapping of pristine **a**) NRM and **b**) V-NRM particles

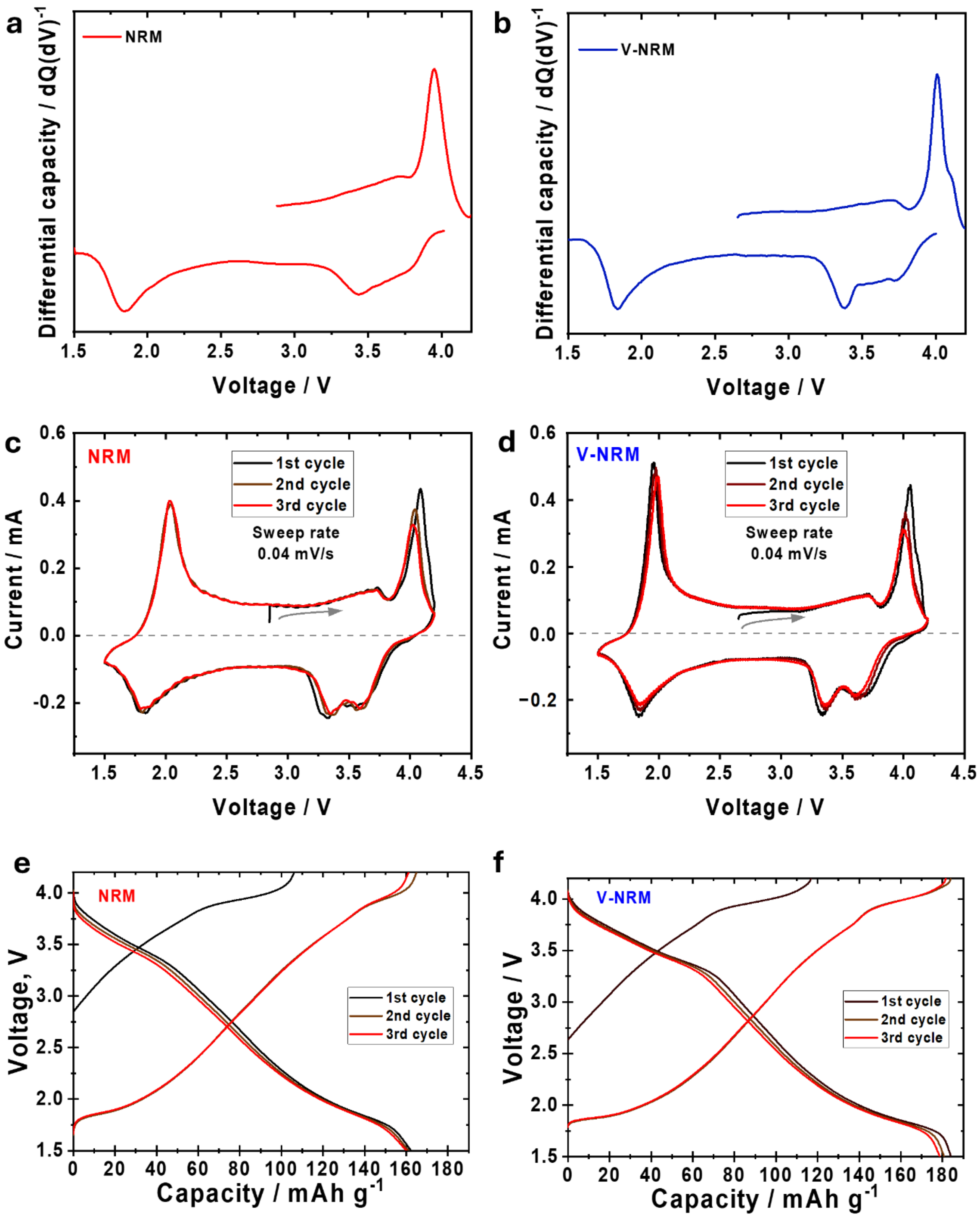

Fig. S2 Comparison of differential capacity (dQ/dV) curves of initial cycles of **a**) NRM and **b**) V-NRM half-cells at 0.05C. Cyclic voltammetry (CV) results of **c**) NRM and **d)** V-NRM half-cells at 0.04 mV/s. Voltage profile results of **e**) NRM and **f**) V-NRM half-cells at 0.1C

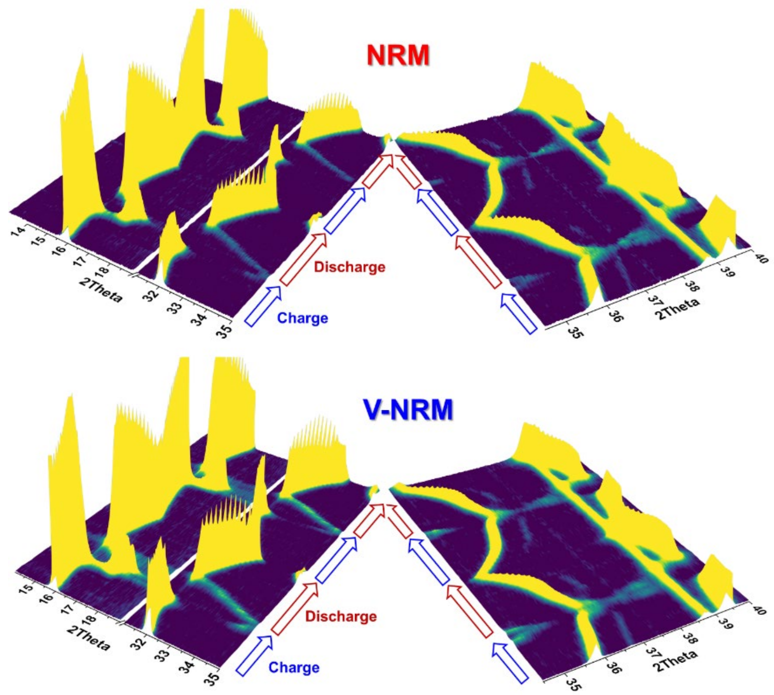

Fig. S3 NRM and V-NRM half-cells comparing operando XRD results from **Fig.** 4 remapped in 3D format

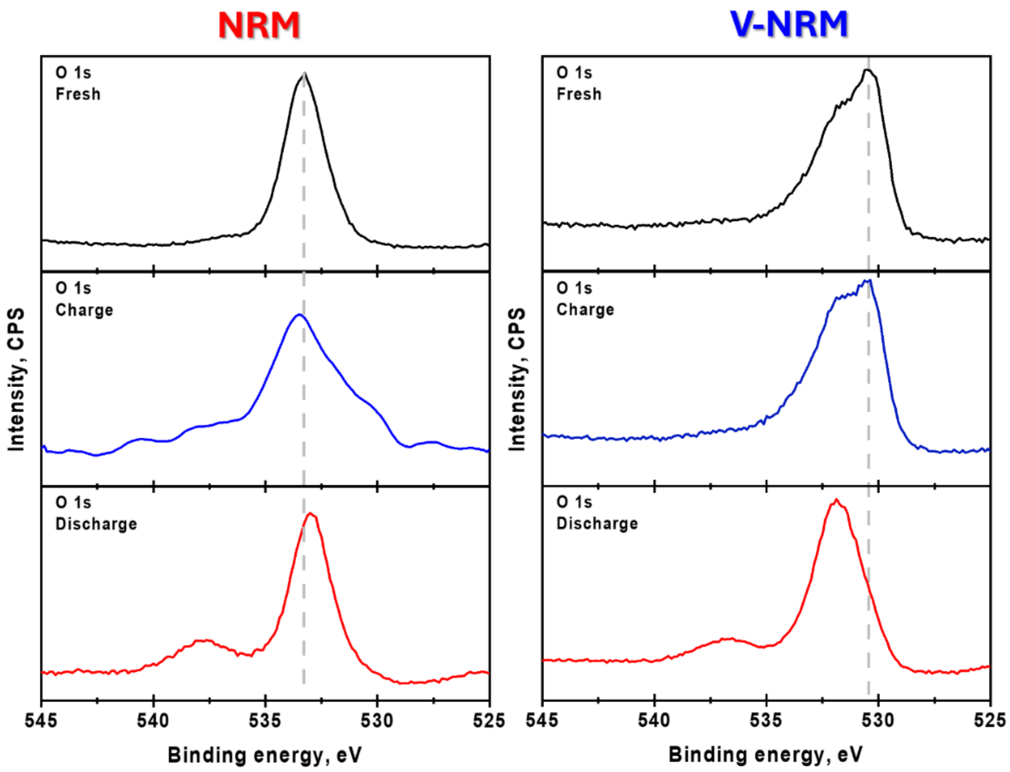

Fig. S4 Comparison of XPS spectra of O 1s of NRM and V-NRM at pristine, charge and discharge states

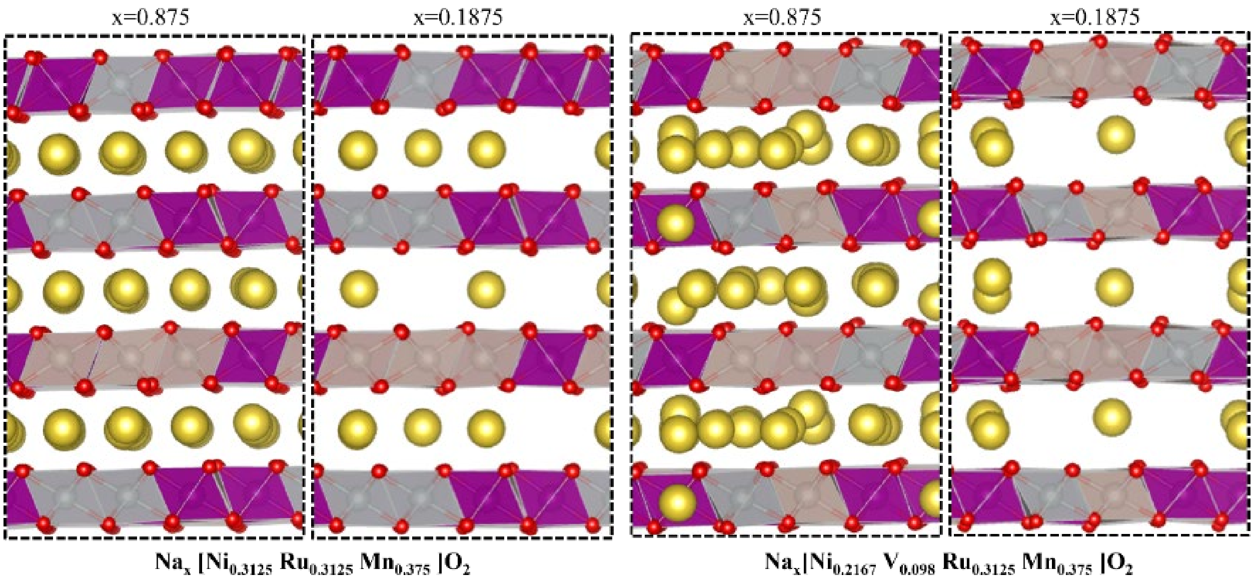

Fig. S5 Computed atomistic structures of $Na_x[Ni_{0.3125}Ru_{0.3125}Mn_{0.375}]O_2$ and $Na_x[Ni_{0.2167}V_{0.098}Ru_{0.3125}Mn_{0.375}]$ O_2 at discharged (x=0.875) and charged (x=0.1875) states
